# A Scale Factor Calibration Method for MEMS Resonant Accelerometers Based on Virtual Accelerations

**DOI:** 10.3390/mi14071408

**Published:** 2023-07-12

**Authors:** Zhaoyang Zhai, Xingyin Xiong, Liangbo Ma, Zheng Wang, Kunfeng Wang, Bowen Wang, Mingjiang Zhang, Xudong Zou

**Affiliations:** 1 The State Key Laboratory of Transducer Technology, Aerospace Information Research Institute, Chinese Academy of Sciences, Beijing 100190, China; 2 School of Electronic, Electrical and Communication Engineering, University of Chinese Academy of Sciences, Beijing 100049, China; 3 QiLu Aerospace Information Research Institute, Jinan 250101, China; 4 Key Laboratory of Advanced Transducers and Intelligent Control System of Ministry of Education, Taiyuan University of Technology, Taiyuan 030024, China

**Keywords:** accelerometer, calibration, virtual accelerations, comb-drive actuators

## Abstract

This paper presents a scale factor calibration method based on virtual accelerations generated by electrostatic force. This method uses a series of voltage signals to simulate the inertial forces caused by the acceleration input, rather than frequent and laborious calibrations with high-precision instruments. The error transfer model of this method is systematically analyzed, and the geometrical parameters of this novel micromachined resonant accelerometer (MRA) are optimized. The experimental results demonstrate that, referring to the traditional earth’s gravitational field tumble calibration method, the error of the scale factor calibration is 0.46% within ±1 g by using our method. Moreover, the scale factor is compensated by virtual accelerations. After compensation, the maximum temperature drift of the scale factor decreases from 2.46 Hz/g to 1.02 Hz/g, with a temperature range from 40 °C to 80 °C.

## 1. Introduction

MEMS accelerometers have been widely used in consumer electronics, industrial control, energy exploration, and aerospace fields due to their small size, low cost, low power consumption, easy integration, and massive production [1,2]. Compared to piezoresistive [3], piezoelectric [4,5], and capacitive [6] accelerometers, micromachined resonant accelerometers (MRAs) have the characteristics of high sensitivity, large dynamic range, and strong anti-interference ability [7,8]. Therefore, they have a good application prospect.

However, the actual performances of MEMS sensors often have a large gap between their theoretical characteristics due to uncertainty in process variation and characterization of the material used in MEMS devices [9,10]. Therefore, it is necessary to calibrate the sensors at the end of the manufacturing process to correct the systematic error of the production process [11]. For MEMS accelerometers, the traditional calibration methods include earth’s gravitational field tumble experiments, precision centrifugal experiments, and shaking table experiments [12,13,14]. These calibration methods rely on sophisticated laboratory equipment, such as the high-precision tilt table and centrifuge. Nevertheless, the environmentally dependent errors [15] (temperature, pressure) or ageing [16] are not able to be corrected by in-factory calibration. These issues result in the drifts of bias and scale factor of MEMS accelerometers during their lifetime. In high-end applications, such as navigation, these drifts cannot be negligible [17]. Therefore, a periodical recalibration of MEMS accelerometers during its lifetime is needed to suppressed the drawback of the time-varying drifts.

Generally, one of the recalibration methods is the disassembling of MEMS accelerometers from the working device and calibration of the laboratory. This method is inconvenient and costly. Therefore, it is necessary to periodically recalibrate accelerometers in the field to obtain high performances during their lifetime. By fixing the accelerometer in a machined housing, the accelerometer is calibrated using the multi-position rotation based on gravity, which escapes the limitations of high-precision laboratory instruments [18,19,20,21]. Frosio proposed a calibration method using at least nine random positions without using a high-precision housing [22]. However, in some special application scenarios, e.g., space and underwater, this recalibration may be impracticable. In previous studies, a micro-vibration platform based on piezoelectric materials has been developed to realize calibration in the field for inertial sensors [23,24,25]. Based on the micro-vibration platform, Li proposed a system that integrates an optical detection sensor for the calibration of accelerometers [26]. In this method, the inertial sensor is fixed on the micro-vibration platform, and the micro-vibration platform provides on-chip physical stimulation. However, the fabrication process of the micro-vibration platform is extremely complicated. In addition, the polarization degradation of piezoelectric materials can affect the motion behavior of the mechanical structure, resulting in the reduction of long-term stability. In recent years, some parameter extraction methods for capacitive accelerometers have been proposed. Parameters such as pull-in voltage and resonant frequency can be extracted through electrostatic excitation, and the scale factor can be indirectly calculated according to the identified parameters. However, the calibration error of this method is 5% [27,28]. There is also a study based on the multi-variate linear regression method to fit the relationship between the output signal amplitude-frequency response, phase-frequency response, and scale factor. By this method, 0.55% rms error in scale factor prediction is achieved. In order to improve the calibration accuracy, this method needs a large number of sample data for the learning purposes [29]. Heringhaus proposed a transfer learning method that reduces the time consumed by parameter fitting in this method [30]. Although the above methods based on electrostatic extraction can identify the scale factor, the quadratic and cubic nonlinearity have not been analysed.

In this paper, a scale factor calibration method based on virtual accelerations for MRAs is proposed. The calibration error transfer model of this method is systematically analysed, including the DC voltage error and non-ideal electric field. Based on this model, the geometric parameters of the MRA are optimized. Finally, the method is verified based on an MRA prototype device. The experimental results demonstrate that, compared with the traditional earth’s gravitational field tumble calibration method, the error of the scale factor does not exceed 0.46% calibrated by the virtual accelerations within ±1 g. Moreover, virtual accelerations are used to compensate for temperature drifts of the scale factor.

## 2. Theoretical Analysis of Self-Calibration Method

### 2.1. Principle of Self-Calibration Method

**Figure 1:**
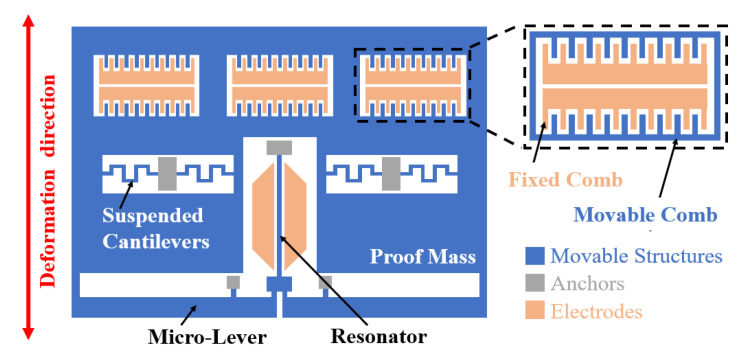
The schematic diagram of the MRA.

The main structures of the MRA include resonator, micro-lever, proof mass, comb-drive actuators, and support structures. The schematic diagram of the MRA is shown in Figure 1. The proof mass is connected to the double-ended fixed beam resonator through a micro-lever force amplifier structure. When there is an external acceleration input, the proof mass will generate an inertial force. The inertial force is amplified by the micro-lever and loaded onto the resonator, causing the frequency shift of the resonator. Therefore, the external acceleration can be calculated by measuring the change of the resonant frequency. In addition, six groups of back-to-back comb-drive actuators generate several electrostatic forces to drive the proof mass. When the voltage is applied to comb-drive actuators, the double-ended fixed beam is under tension or compression. This also changes the resonant frequency of the resonator. Therefore, electrostatic forces can be used to calibrate the accelerometer.

The vibration equation of the proof mass can be written as a second-order mass-damper-spring system [31]:

(1) $$m\ddot{x}\left(t\right)+b\dot{x}\left(t\right)+kx=ma+F_{e}$$

where *m* is the mass of the proof mass, *b* is the damping coefficient, *k* is the sum of spring stiffness of the suspended cantilever and micro-lever force amplifier structure, *x* is the displacement of the proof mass, *a* is the input physical acceleration, and $F_{e}$ is the electrostatic force generated by comb-drive actuators.

The MRA is sealed in a vacuum package with negligible damping, and the bandwidth of the input acceleration is usually much lower than the resonant frequency of the second-order system. Therefore, Equation (1) can be simplified as:

(2) $$kx=ma+F_{e}$$

Both electrostatic and inertial forces cause displacement of the proof mass. We assume that the electrostatic force generates a virtual acceleration. The structures used here to generate electrostatic forces are comb-drive actuators rather than parallel plate actuators [32]; ideally, the voltage and virtual acceleration are linear. Therefore, the relationship between the DC voltage and the virtual acceleration can be noted:

(3) $$a_{v}=\frac{F_{e}}{m}=k_{VA}{V_{dc}}^{2}$$

where $a_{v}$ is the virtual acceleration, $V_{dc}$ is the DC voltage, and $k_{VA}$ is the conversion coefficient between the virtual acceleration and the DC voltage. Different virtual accelerations can be obtained by changing the DC voltage.

**Figure 2:**
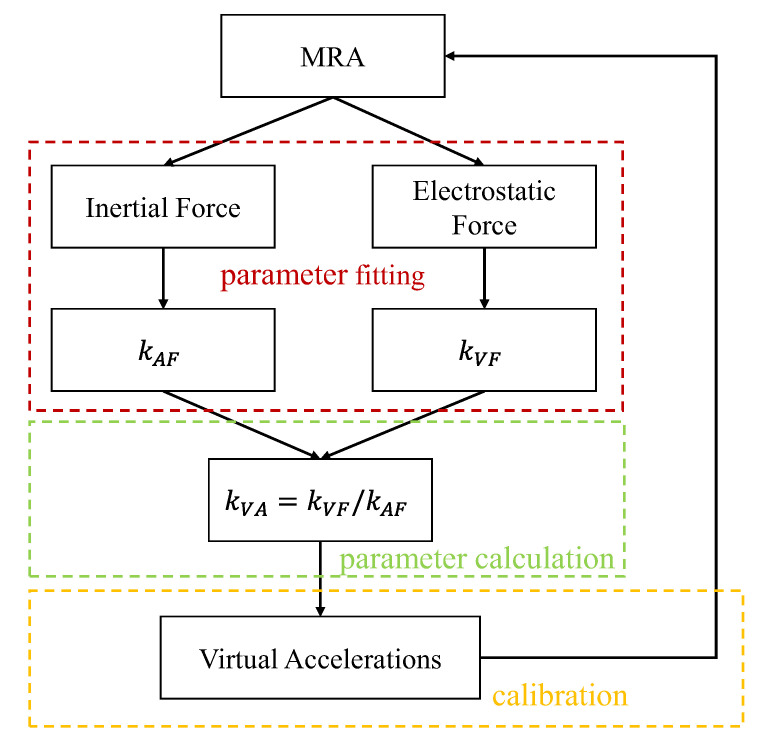
Block diagram of the online calibration method.

The calibration method based on virtual accelerations is divided into the following steps, as shown in Figure 2. Firstly, the conversion coefficient $k_{AF}$ between physical acceleration and resonant frequency can be obtained by applying gravitational force to the MRA by the high precision tilt table. Then, the conversion coefficient $k_{VF}$ between the DC voltage and resonant frequency can be obtained by applying electrostatic force to MRA. After further calculation, $k_{VA}=k_{VF}/k_{AF}$ is obtained. Finally, comb-drive actuators generate different virtual accelerations to realize calibration in some inconvenient disassembly scenarios.

### 2.2. Calibration Error Transfer Model

The static input-output model of MRA is described as [33]:

(4) $$E=K_{0}+K_{1}a_{i}+K_{2}{a_{i}}^{2}+K_{3}{a_{i}}^{3}$$

where E is the output signal of MRA, and $K_{0}$, $K_{1}$, $K_{2}$, $K_{3}$ are bias, scale factor, quadratic, and cubic nonlinearity, respectively. During the calibration process, a set of accelerations $a_{i}$(i=1,2,3,…,n) is generated by the comb-drive actuators, and the output $E_{i}$(i=1,2,3,…,n) is measured simultaneously. Then, K is calculated by the least square method.

(5) $$K={\left(A^{T}A\right)}^{-1}A^{T}E$$

where

$$\left\{\begin{array}{c} K=\left[K_{0}\;K_{1}\;K_{2}\;K_{3}\right]\;\;\\ {E=[E_{1}\;E_{2}\ldots E_{n}]}^{T}\\ A=\left[\begin{array}{c} \begin{array}{c} 1\;\;\;a_{1}\;\;\;a_{1}^{2}\;\;\;a_{1}^{3}\\ 1\;\;\;a_{2}\;\;\;a_{2}^{2}\;\;a_{2}^{3} \end{array}\\ \ldots \;\;\;\ldots \;\;\;\ldots \\ 1\;\;\;a_{n}\;\;\;a_{n}^{2}\;\;\;a_{n}^{3} \end{array}\right] \end{array}\right.$$

In fact, virtual acceleration is not ideal. Considering the non-ideal factors, the input virtual acceleration $a_{v}$ is detailed as:

(6) $$a_{v}=a_{l}\left(1+\lambda_{dc}+\lambda_{non}a_{l}\right)$$

where $a_{l}$ is the virtual acceleration generated by the ideal comb-drive actuators, $\lambda_{dc}$ is the error caused by DC voltage, and $\lambda_{non}$ is the error caused by the electrostatic nonlinearity. The derivation of each coefficient will be described in detail in Section 2.3 and Section 2.4.

By substituting Equation (6) into Equation (4), the static input–output model of MRA with error terms is described as:

(7) $$E=K_{0}+K_{1}a_{l_{i}}\left(1+\lambda_{dc}+\lambda_{non}a_{l_{i}}\right)+K_{2}{a_{l_{i}}}^{2}{\left(1+\lambda_{dc}+\lambda_{non}a_{l_{i}}\right)}^{2}+K_{3}{{a_{l_{i}}}^{3}\left(1+\lambda_{dc}+\lambda_{non}a_{l_{i}}\right)}^{3}$$

Therefore, the bias, scale factor, quadratic, and cubic nonlinearity calibrated by virtual accelerations are $K_{0}^{\prime },\;K_{1}^{\prime },\;K_{2}^{\prime }$, and $K_{3}^{\prime }$, respectively.

(8) $$\left\{\begin{array}{c} K_{0}^{\prime }=K_{0}\\ K_{1}^{\prime }=K_{1}\left(1+\lambda_{dc}\right)\\ K_{2}^{\prime }=K_{2}\left(1+2\lambda_{dc}\right)+K_{1}\lambda_{non}\\ K_{3}^{\prime }=K_{3}(1+{3\lambda }_{dc})+2K_{2}\lambda_{non} \end{array}\right.$$

In Equation (8), the influence of some small terms is ignored. It indicates that $K_{0}^{\prime }$ is not affected by error terms and $K_{1}^{\prime }$ is affected by the DC voltage accuracy. Additionally, $K_{2}^{\prime }$ is related to the product term of $K_{1}\lambda_{non}$ and $2\lambda_{dc}$. The $K_{3}^{\prime }$ is related to the product term of ${2K}_{2}\lambda_{non}$ and $3\lambda_{dc}$. By analysing the source of these error terms, the calibration error can be reduced to a reasonable range.

### 2.3. Influence of DC Voltage Accuracy

Actually, the accuracy of the DC voltage source is limited. By substituting the DC voltage containing the error term into Equation (3) and simplifying it, we obtain:

(9) $$a_{v}=a_{l}(1+\frac{2V_{dce}}{V_{dcl}})$$

We note $\lambda_{dc}=2V_{dce}/V_{dcl}$. For the scale factor, to achieve a calibration accuracy of 1%, the relative error of DC voltage source cannot exceed 0.5%.

### 2.4. Influence of Non-Ideal Electric Field

**Figure 3:**
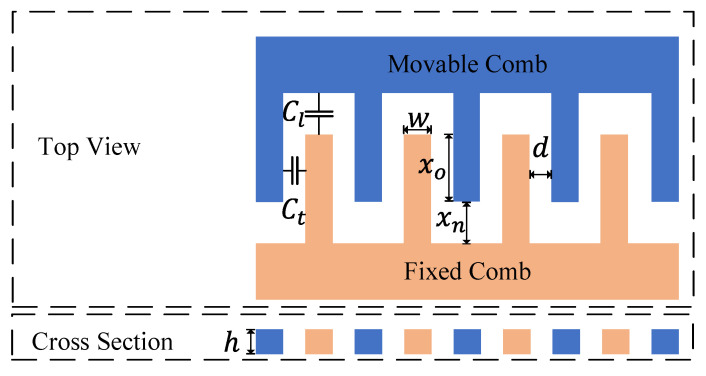
The schematic diagram of the comb-drive actuator.

There are tangential and lateral electric field in comb-drive actuators, as shown in Figure 3 [34]. $C_{t}$ is the tangential capacitance, $C_{l}$ is the lateral capacitance, *h*, *d*, $x_{o}$, $x_{n}$, and *w* are thickness, gap, overlap length, non-overlap length, and width of the comb-drive actuator, respectively.

The tangential and lateral capacitance of the comb-drive actuators are obtained by [35]:

(10) $$C_{t}=2Nn\frac{\varepsilon (x_{o}+x)h}{d}$$

(11) $$C_{l}=N(2n+1)\frac{\varepsilon hw}{x_{n}-x}$$

where *N* is number of comb groups, *n* is number of combs in each group, $\varepsilon =8.854\times 10^{-12}\;F/m$ is the air electrical permittivity, and *x* is the displacement of the proof mass. The electrostatic force is obtained by:

(12) $$F_{e}=\frac{1}{2}(\frac{\partial C_{t}}{\partial x}+\frac{\partial C_{l}}{\partial x})V_{dc}^{2}$$

(13) $$\frac{\partial C_{t}}{\partial x}=2Nn\frac{\varepsilon h}{d}=\alpha$$

(14) $$\frac{\partial C_{l}}{\partial x}=N\left(2n+1\right)\frac{\varepsilon hw}{{x_{n}}^{2}}+2N\left(2n+1\right)\frac{\varepsilon hw}{{x_{n}}^{3}}x=\beta +\gamma x$$

The x is usually on the order of $10^{-7}$ m, while $x_{n}$ is usually on the order of $10^{-5}$ m, since $x\ll x_{n}$, ${\partial C}_{l}/\partial x$ is expanded into Taylor series and neglected the higher order terms. According to Equations (3) and (12), the virtual acceleration considering electrostatic nonlinearity can be described as:

(15) $$a_{v}=a_{l}\left(1+\frac{\gamma }{\alpha +\beta }x\right)=a_{l}\left(1+\frac{\gamma }{\alpha +\beta }\frac{ma_{v}}{k}\right)$$

We note $\lambda_{non}=\frac{\gamma }{\alpha +\beta }\frac{m}{k}$. By expanding the Equation (15) and ignoring the small terms, we observe:

(16) $$a_{v}=a_{l}(1+\lambda_{non}a_{d})$$

**Figure 4:**
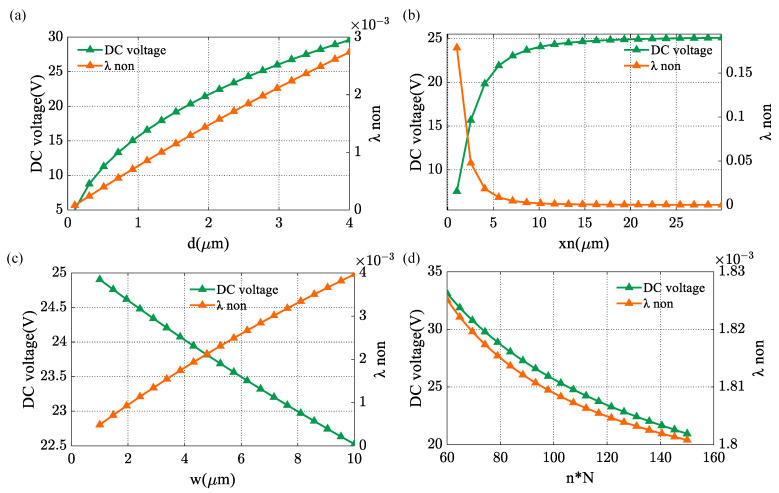
The effect of structural parameters on DC voltage and $\lambda_{non}$. (**a**) gap from 100 nm to 4 μm; (**b**) non-overlap length from 1 μm to 30 μm; (**c**) width from 1 μm to 10 μm; and (**d**) N∗n from 60 to 150.

The effect of structural parameters on the DC voltage used to generate 1 g acceleration and $\lambda_{non}$ are shown in Figure 4.

According to the results, the smaller gap of comb-drive actuators, the smaller of DC voltage, and $\lambda_{non}$ will be limited by the fabrication process tolerance. With the reduction of the non-overlap length, the DC voltage will decrease, but it will increase the $\lambda_{non}$. Increasing width will increase the $\lambda_{non}$ and reduce the DC voltage. Increasing n∗N will reduce the DC voltage and $\lambda_{non}$, but it increases the difficulty of the fabrication process and decrease proof mass. The effect of overlap length is not reflected in our analysis, but in order to reduce the difficulty of the process, the overlap length should not be too large. We do not discuss the effect of thickness here because it affects not only the comb-drive actuators, but also the micro-lever and suspended cantilevers that have been designed. Based on these analyses, the structural parameters of the MRA prototype device are summarized in Table 1. We can get results as $k_{VA}=1.73\times 10^{-3}\;g/V^{2}$, the DC voltage required to generate 1 g of virtual acceleration is 24.03 V and $\lambda_{non}=0.18\%$.

## 3. Design and Fabrication

**Figure 5:**
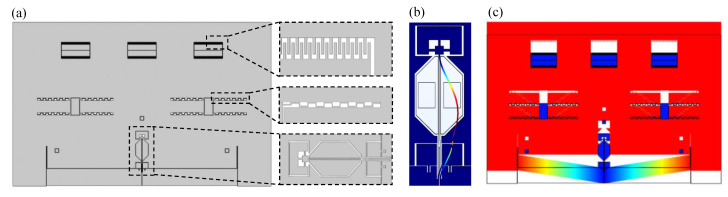
(**a**) The finite element method (FEM) simulation model of the MRA; (**b**) The first vibration mode of the resonator; (**c**) The deformation of MRA.

The finite element method (FEM) simulation model (using COMSOL 5.6) of the MRA is shown in Figure 5a. Figure 5b shows the first vibration mode of the resonator with a resonant frequency of 185.875 kHz.

In the FEM, the displacement of the proof mass can be directly extracted, and the deformation is shown in the Figure 5c. Therefore, the $k_{VA}$ can be calculated according to the displacement. By changing the acceleration, the response curve of acceleration and displacement can be obtained. The coefficient between acceleration and displacement is $k_{Ax}=9.95\times 10^{-8}\;m/g$. By changing the DC voltage, the voltage and displacement response curve can be obtained. The coefficient between the voltage and the displacement is $k_{Vx}=1.19\times 10^{-10}\;{m/V}^{2}$. Then, $k_{VA}=k_{Vx}/k_{Ax}=1.196\times 10^{-3}\;g/V^{2}$ can be obtained. The finite element simulation results verify the feasibility of this calibration method. In the FEM simulation model, it is found that the mesh accuracy has a great influence on the electrostatic force.

**Figure 6:**
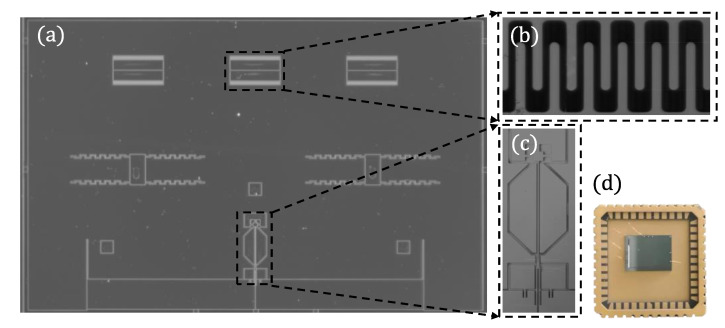
(**a**) The microscope image of MRA; (**b**) Back-to-back comb-drive actuators; (**c**) The sensing electrode, driving electrode, and double-ended fixed beam of the resonator; and (**d**) The MRA is fixed on the chip carrier by adhesive.

The MRA prototype device is fabricated using the Silicon-On-Insulator (SOI) process and wafer-level vacuum package. The microscope image is shown in Figure 6a. There are six groups of back-to-back comb-drive actuators, and its edges are designed into a circle to avoid tip discharge as shown in Figure 6b. The resonator consists of sensing electrode, driving electrode and double-ended fixed beam as shown in Figure 6c. The MRA prototype device is fixed on the chip carrier by adhesive, as shown in Figure 6d.

## 4. Experiments and Results

**Figure 7:**
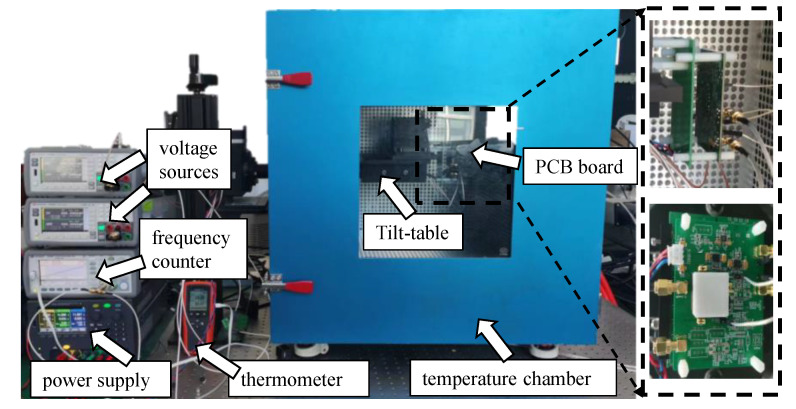
Test environment setup.

To verify the calibration method based on virtual acceleration, we set up the test environment as shown in Figure 7. The MRA was mount on a tilt-table with a positioning accuracy of $1^{\prime }$ for applying the physical acceleration. Two low-noise voltage sources (Keysight B2962A) are used to supply $V_{dc1}$ and $V_{dc2}$. Different virtual accelerations are obtained by adjusting $V_{dc1}$ and $V_{dc2}$. The resonant frequency of the accelerometer is characterized by a frequency counter (Keysight 53230A). The temperature chamber is used to change the ambient temperature and is monitored through a thermometer.

**Figure 8:**
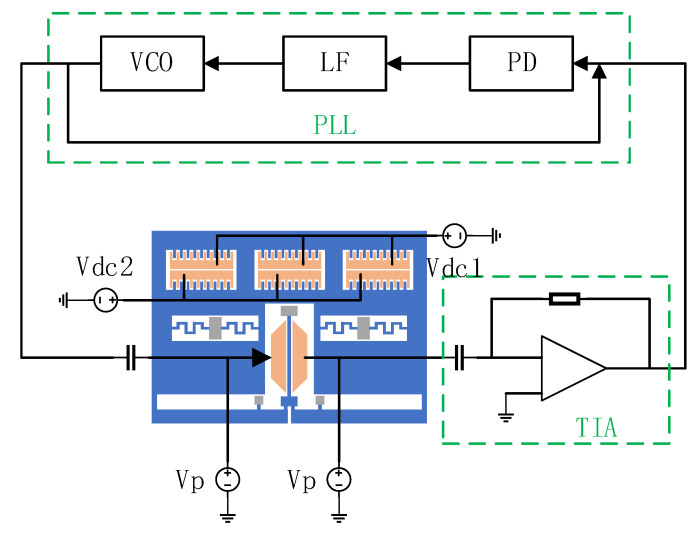
The schematic diagram of the test circuit.

The schematic diagram of the test circuit is shown in Figure 8. The motional current of the MRA is first converted into a voltage signal through a transimpedance amplifier (TIA). The closed loop circuit is based on the Phase-Locked Loop (PLL) [36]. The phase detector (PD) detects the phase difference. Then, the phase difference signal is passed through the loop filter (LF) to form the control voltage of the voltage-controlled oscillator (VCO). Finally, the VCO adjusts the frequency of the driving signal. When the resonant frequency changes, the PLL can track its change.

Follow the calibration process shown in Figure 2. By adjusting the $V_{dc1}$ and $V_{dc2}$ to 0 V and adjusting the tilt-table to apply ±1 g physical acceleration to the MRA, the $k_{AF}=841.68\;\mathrm{Hz}/g$ is obtained. Then, adjust the tilt table to make the physical acceleration to 0g and the DC voltage source to apply 10 V voltage to comb-drive actuators, respectively. The $k_{VFn}=1.062\;\mathrm{Hz}/V^{2}$ and $k_{VFp}=1.05\;\mathrm{Hz}/V^{2}$ is obtained, so we can obtain $k_{VAn}=1.26\times 10^{-3}\;g/V^{2}$ and $k_{VAp}=1.24\times 10^{-3}\;{g/V}^{2}$. $k_{VAn}$ and $k_{VAp}$ are coefficients for the back-to-back comb-drive actuators. Due to process tolerances, the coefficients of the comb-drive actuators that generate virtual acceleration in two opposite directions are different.

**Figure 9:**
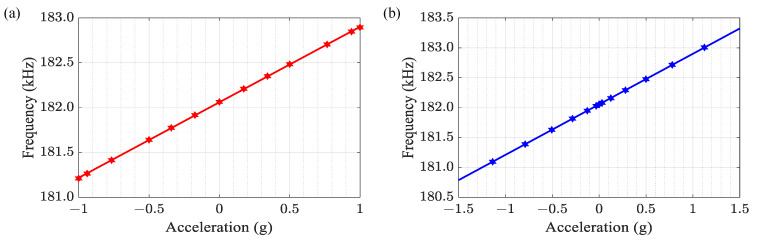
(**a**) The physical acceleration and frequency curve; (**b**) The virtual acceleration and frequency curve.

We used two methods to calibrate the MRA. Based on the traditional tilt-table calibration method, the frequency output of the MRA is shown in Figure 9a. The scale factor calibrated by the tilt-table is 842.1 Hz/g. Then, the virtual accelerations are generated when the voltages applied to the comb-drive actuators are 0 V, 5 V, 10 V, 15 V, 20 V, 25 V, and 30 V, and the frequency output of the MRA is shown in Figure 9b. The scale factor calibrated by virtual accelerations of 846.01 Hz/g. We assume that the scale factor calibrated by the tilt-table have sufficient accuracy as the reference value. Referring to the traditional tilt-table calibration, the calibration error of the scale factor using our methods is 0.46%.

**Figure 10:**
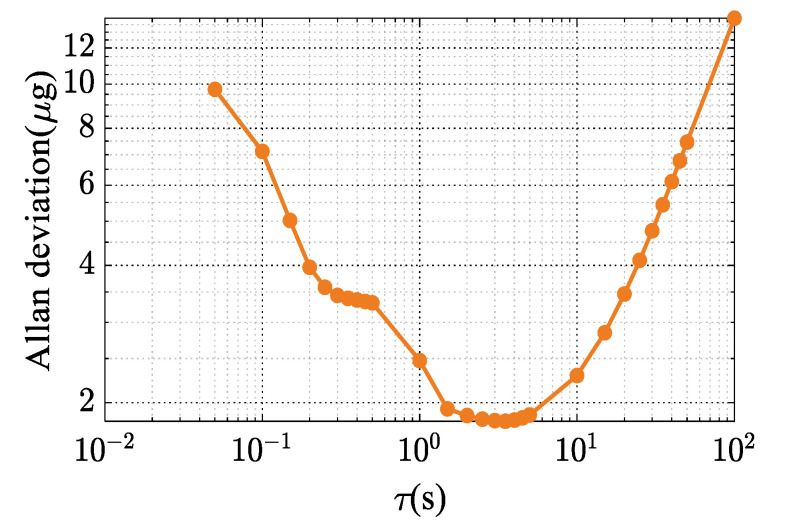
Experimental measured results of the Allan deviation.

Then, we tested the bias instability of the MRA, as shown in Figure 10. Allan deviation shows that the bias instability is fewer than 2 μg. Therefore, the maximum calibration error caused by the bias instability does not exceed 0.003 Hz/g. Additionally, it can be presumed that the maximum error in the scale factor due to positioning error of the tilt-table is 0.48 Hz/g. These results indicate that the calibration error of 0.46% is mainly generated by this calibration method rather than measurement errors.

**Figure 11:**
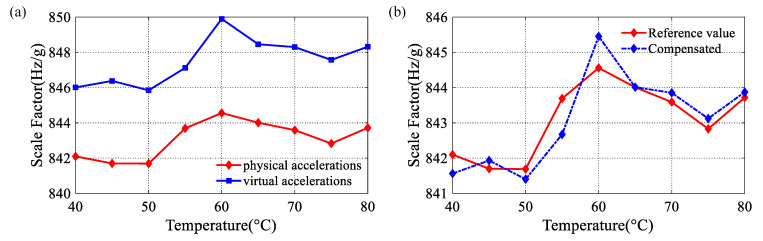
(**a**) The scale factor calibrated by virtual accelerations and physical accelerations at different temperatures; (**b**) Compensation of scale factor based on virtual accelerations.

Additionally, we calibrate the scale factor using two methods at different ambient temperatures. The results are shown in Figure 11a. The calibration error obtained is between 0.41% and 0.66%, which indicates that the error is relatively stable. It proves that the calibration method based on virtual acceleration has good temperature robustness. Therefore, the scale factor drift caused by temperature can be compensated for based on the calibration results of virtual accelerations. The compensation process is as follows: Firstly, we take the difference between the calibration results of the two methods. Then, we take the average of the differences. Finally, we subtract the average value from the scale factor calibrated by the virtual accelerations. The result after compensation is shown in Figure 11b. The scale factor calibrated by physical accelerations at 40 °C is called the uncompensated value. The maximum error relative to reference value at each temperature is 2.46 Hz/g, and the average error is 1.18 Hz/g. However, the maximum error after compensation is 1.02 Hz/g, and the average error is 0.41 Hz/g. The online compensation of the scale factor can be realized by further optimization of the measurement and control circuit.

## 5. Conclusions

In this paper, a method of the scale factor calibration for MRAs is proposed based on virtual accelerations. This method avoids frequent use of high-precision instruments for recalibration of the accelerometers, which can be used as a scheme for field calibration of the accelerometer. The calibration accuracy is systematically analyzed including the error of DC voltage and the electrostatic nonlinearity of the comb-drive actuators. The feasibility of the method is verified by FEM and the MRA is designed, fabricated, and tested. The experimental results show that the calibration result of scale factor based on our method is 846.01 Hz/g, referring to the traditional tilt-table calibration method, the normalized error is 0.46% within ±1 g. Furthermore, we compensate for scale factor drift caused by temperature based on virtual accelerations. A possible application scenario for this method is to compensate for the scale factor thermal drift under variable acceleration measurements.

Compared to the method of earth’s gravitational field tumble experiments, after the first calibration is completed, this calibration method can be used even if there is no standard gravity reference. Moreover, compared to a micro-vibration platform, this method has a simpler structure and does not require additional manufacturing processes. This method can be applied to other MRA designs by adding comb-drive actuators to the original MRA structure. In addition, low dropout regulator (LDO) can be used to replace high-precision voltage sources. In future work, an automatic calibration system of MRA will be researched based on this calibration method. It mainly includes the automatic generation of virtual accelerations and the automatic acquisition and processing of the MRA output signal through microprocessor.

## Figures and Tables

**Table 1:** The structural parameters of the MRA.

Parameter	Variable	Value
Width	w	4 μm
Thickness	h	50 μm
Gap	d	2.5 μm
Overlap length	$x_{o}$	23 μm
Non-overlap length	$x_{n}$	10 μm
Number of combs in each group	n	38
Number of comb groups	N	3
Proof mass	m	1.31 mg
Spring stiffness	k	130.7 N/m

## Data Availability

Not applicable.
